# Relationships Among Psychological Risk, Eco-Friendly Packaging, Price Fairness, and Brand Trust of Bottled Water Consumers: Moderating the Impact of Nutritional Disclosure

**DOI:** 10.3390/foods13233800

**Published:** 2024-11-26

**Authors:** Kyung-A Sun, Joonho Moon

**Affiliations:** 1 Department of Tourism Management, Gachon University, Sungnam-si 13120, Republic of Korea; kasun@gachon.ac.kr; 2 Department of Tourism Administration, Kangwon National University, Chuncheon 24341, Republic of Korea

**Keywords:** bottled water, psychological risk, price fairness, brand trust, nutritional disclosure

## Abstract

This study explores the relationship between psychological risk, price fairness, and brand trust in consumers of bottled water. We also tested the moderating effect of nutritional disclosure on the impacts of psychological risk and eco-friendly packaging on price fairness. We analyzed the data of 308 participants recruited via the Clickworker platform. Hayes’ PROCESS macro model 7 was employed to test the hypotheses. Price fairness was negatively influenced by psychological risk. Moreover, brand trust was significantly impacted by psychological risk and price fairness, with a significant moderating effect of nutritional disclosure on the relationship between eco-friendly packaging and price fairness. This work adds to the literature by identifying the relationship among four factors relevant to bottled water businesses.

## 1. Introduction

Grand View Research [1] noted that the US bottled water market was worth approximately USD 39.3 billion in 2022. Many companies have been competing in this market. According to Statista [2], there are more than 10 major players in the bottled water market. It is essential to identify consumer characteristics to manage constrained resources effectively. Therefore, this research examines consumer behavior in the context of bottled water, focusing on US consumers. To examine consumer behavior, this research uses brand trust as the main attribute, because the bottled water business uses the brand for distinction in the market [3]. That is, brand trust is the dependent variable because many studies have found that brand trust is key to increasing sales by fostering loyalty in consumers [4,5,6]. 

This study similarly focuses on price fairness. Price refers to what a consumer pays to attain certain goods or services [7,8]. An affordable price is critical for sales growth, and consumers perceive “rational costs” as indicative of price fairness [9,10]. Price fairness appears to be a major factor in consumer behavior. Psychological risk refers to the degree of uncertainty associated with purchasing goods or services [11,12]. Many studies have scrutinized the influence of psychological risk on the appraisal of goods and services [11,13,14]. Here, we studied psychological risk in the context of consumer behavior concerning bottled water. Moreover, we investigated the role of perceived eco-friendliness, which is related to the amount of plastic waste, because concern regarding the environment continues to grow because of growing concern in the market about protecting the natural environment [15,16]. In summary, we examined consumer behavior about bottled water products, exploring the roles of brand trust (the dependent variable), price fairness (the mediator), psychological risk, and eco-friendly packaging (the independent variables). 

This work also examined the moderating role of nutritional disclosure—specifically, the provision of nutritional information to consumers [17,18]. This information plays a crucial role in consumer decision-making by reducing uncertainty and providing transparency [17,19]. Given that consumer behaviors can vary significantly, we investigated whether product-related information, such as nutritional disclosure, influences the relationship between psychological risk, eco-friendly packaging, and price fairness.

The primary objective of this study is to explore the interrelationships between brand trust, price fairness, psychological risk, and eco-friendly packaging. By doing so, we aim to contribute to the literature on consumer behavior, particularly in the context of bottled water, by clarifying how these factors interact. Another key objective is to assess how nutritional disclosure moderates the impact of psychological risk and eco-friendly packaging on price fairness. This investigation provides valuable theoretical insights by elucidating these relationships. Our findings offer important managerial implications for the bottled water industry, enhancing the understanding of consumer preferences and decision-making processes in this sector.

## 2. Literature Review and Hypothesis Development

### 2.1. Psychological Risk

Psychological risk refers to a negative viewpoint of consumers [11,12], where goods may be perceived as not capable of satisfying needs [13,14,20]. Many studies have explored psychological risk in various contexts. Öztürk [12] examined it among consumers of halal products, while Lăzăroiu et al. [20] explored it among users of social commerce platforms. Seo and Lee [13] investigated perceived risk among users of service robots.

### 2.2. Eco-Friendly Packaging

Prior studies on eco-friendly packaging have focused on minimizing waste and environmental pollution [21,22]. Galati et al. [23] reported that interest in eco-friendly packaging is increasing because of the growing problem of global warming. Koch et al. [16] argued that such packaging is important because consumers link it with social issues. Nguyen et al. [24] found that consumers are more likely to choose products with eco-friendly packaging. Finally, Ketelsen et al. [15] and Yan et al. [25] reported that eco-friendly packaging is critical to food businesses because foods with such packaging are favored by consumers. Hence, the use of eco-friendly packaging is an important market trend.

### 2.3. Price Fairness

Price fairness refers to consumer perceptions of the prices set by vendors [7,8]. It is essential for success in business because it is related to first impressions and the general consumer experience. Singh et al. [10] scrutinized the price-fairness perceptions of fast-food restaurant customers, while Jung et al. [9] studied the phenomenon in apparel product consumers. Bolton et al. [7] researched differences in price-fairness perceptions between domestic and international consumer behavior.

### 2.4. Brand Trust

Brand trust refers to the perceived credibility of a given brand [4,5]. It is considered a form of reliability established via positive consumption experiences [6,26]. Akoglu and Özbek [5] analyzed brand trust in the context of sports goods consumers, and Sohaib and Han [26] explored its influence in the domain of social media marketing. Na et al. [27] examined it among smartphone consumers. Finally, Lin and Xu [28] explored it in bottled water consumers. 

### 2.5. Hypothesis Development

Studies have found that greater transparency of information lowers uncertainty, significantly impacting perceptions of price fairness [9,29]. Konuk [30] found that price fairness is an essential determinant of consumer behavior concerning organic food. Masoud [31] argued that consumers’ psychological risk negatively affects their perception of price, as the cost becomes more uncertain under unstable conditions, particularly in the context of online shopping. Similarly, Ventre and Kolbe [32] posited that consumers’ risk perceptions lead to a negative impact on decision-making, as they are unsure whether the price is justified for the value of the goods being purchased. The provision of information is likely to improve perceptions of price fairness. A negative influence of risk on trust has been noted in the area of online commerce [33]. Similarly, Ha et al. [34] examined autonomous vehicle consumers and found that psychological risk negatively affected trust. Jun [35] found a significant negative association between psychological risk and brand trust employing Airbnb system users. Zadha and Suparna [36] showed the negative impact of psychological risk on brand trust in digital banking services. Finally, Ali et al. [37] reported a negative impact of risk on trust among fintech users. Against this background, this work proposes the following hypotheses:

**H1a:**  *Psychological risk negatively impacts price fairness*.

**H2a:**  *Psychological risk negatively impacts brand trust*.

Galati et al. [23] found that consumers are willing to pay a premium for bottled water with eco-friendly packaging. Similarly, Koch et al. [16] demonstrated that such packaging enhances consumer perceptions by alleviating environmental guilt. Nguyen et al. [24] reported that Vietnamese consumers also show a willingness to pay more for products with eco-friendly packaging. Additionally, Ketelsen et al. [15] suggested that consumers tend to view eco-friendly packaging more favorably because it is often lighter and perceived as “safer”, reducing concerns about chemical contamination and the misleading effect of heavy packaging on the actual product quantity. Building on these findings, the present study proposes the following hypotheses:

**H1b:**  *Eco-friendly packaging positively impacts price fairness*.

**H2b:**  *Eco-friendly packaging positively impacts brand trust*.

A positive effect of price fairness on trust was noted among social network service users because adequate price is critical to building a positive appraisal of business [38]. Similarly, Hutama and Ekawati [39] found a positive association between price fairness and trust, as did Chubaka Mushagalusa et al. [40] in the domain of financial services. This work proposes the following hypothesis:

**H3:**  *Price fairness positively impacts brand trust*.

### 2.6. Nutritional Disclosure 

Nutritional disclosure refers to the disclosure of the ingredients and nutrients of food products [41,42]. Such information is essential to minimize consumer uncertainty [17,18] and aid in decision-making [17,19]. Therefore, it could be presumed that the nutrition disclosure is likely to minimize the consumers’ negative perception of uncertainty because consumers tend to dislike uncertainty in their decision-making for commercial cases. Hence, the following hypothesis is proposed:

**H4a:**  *Nutritional disclosure significantly moderates the relationship between psychological risk and price fairness*.

Beverage products provide information on volume because of their association with calories [43,44]. Eco-friendly packaging is lightweight because the focus is on minimizing waste [15,25]. Because beverage product nutrition information includes product weight [17,43], heavy packaging may cause confusion among consumers. Therefore, consumers are likely to prefer lightweight packaging for bottled water products because it can minimize suspicion in the step of product purchasing. The final hypothesis of this study is as follows:

**H4b:**  *Nutritional disclosure significantly moderates the relationship between eco-friendly packaging and price fairness*.

## 3. Methods

### 3.1. Research Model

**Figure 1:**
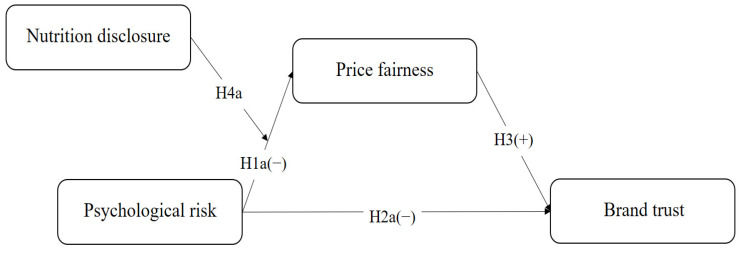
Research model focusing on psychological risk.

Figure 1 shows the first research model used in this study, which has four attributes: psychological risk, price fairness, brand trust, and nutritional disclosure. In the model, the psychological risk is negatively related to price fairness and brand trust; price fairness positively affects brand trust, and nutritional disclosure significantly moderates the relationship between psychological risk and price fairness. 

**Figure 2:**
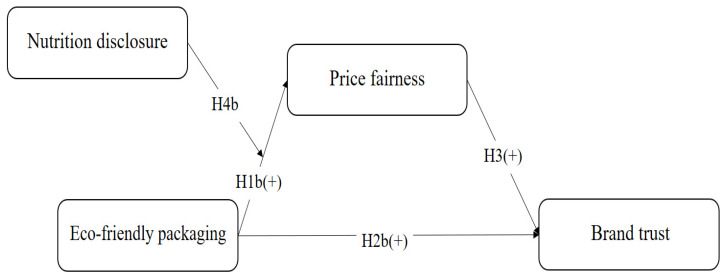
Research model focusing on eco-friendly packaging.

Figure 2 shows the second research model, in which eco-friendly packaging is an independent variable, price fairness is a mediator, and brand trust is the dependent variable. In addition, nutritional disclosure significantly moderates the effect of eco-friendly packaging on price fairness, and all variables are positively associated. 

### 3.2. Measurement Items

Table 1 shows the measurement items in this study. We used a 5-point Likert scale (1 = strongly disagree, 5 = strongly agree) to measure price fairness, nutritional disclosure, and psychological risk and a semantic differential scale to measure brand trust. Measurement items for brand trust [4,5,6], price fairness [7,8,9], nutritional disclosure [17,18,19], psychological risk [12,13,20], and perceptions of eco-friendly packaging [24,45] were derived from the previous literature and modified according to our specific requirements. Brand trust was explored in the context of the “Dasani” brand, as was perceived price fairness. Nutritional disclosure is defined as the information on product ingredients provided to consumers. Also, psychological risk is defined as the difference between consumers’ actual perceptions and their prior expectations regarding Dasani. Perceptions of eco-friendly packaging (packaging focused on protecting the environment) were similarly measured in the specific context of Dasani. 

### 3.3. Recruitment of Participants

This study recruited participants via the Clickworker platform (https://www.clickworker.com, accessed on 23 April 2024), which is commonly used by social science researchers for this purpose [46,47]. The data collection period was 24–27 April 2024. A screening question pertaining to whether the participants were familiar with Dasani was provided; those who were not (35 of 343 respondents) were excluded. Thus, the final number of respondents whose data were analyzed was 308. We focused on Dasani because it has the largest market share in the USA, such that the use rate of Dasani bottled water was deemed likely to be high among the participants. Table 2 displays the demographic information of the participants, including sex, employment, age, and monthly household income. 

### 3.4. Data Analysis

Frequency analysis of the demographic information of the participants was performed, followed by exploratory factor analysis using the Varimax rotation framework. To assess goodness of fit, the following standards were adopted: Kaiser–Meyer–Olkin measure of sampling adequacy > 0.7 and Bartlett’s test of sphericity (χ^2^) [48]. Also, following Hair et al. [48], we adopted the following criteria to evaluate the measurement items’ convergent validity: factor loading > 0.5, Cronbach’s α > 0.7, and eigenvalue > 1. A correlation analysis was conducted to inspect the relationships among variables, with means and standard deviations calculated for brand trust, price fairness, nutritional disclosure, and psychological risk. We also used Hayes’ PROCESS macro model 7, which employs ordinary least squares regression for path analysis. Hayes [49] noted that the PROCESS macro model is less constrained by sample distortion, allowing for more robust estimation. Finally, we performed a simple slope line method to scrutinize the moderating effects of nutrition disclosure. 

## 4. Results

### 4.1. Results of Exploratory Factor Analysis and Reliability Testing

Table 3 shows the results of the exploratory factor analysis. The Kaiser–Meyer–Olkin (0.887) and Bartlett’s test (χ^2^ = 4550.582) statistics were statistically significant. The factor loading and Cronbach’s α values suggested acceptable convergent validity for the measurement items. All five variables have four items. 

### 4.2. Correlation Matrix and Descriptive Statistics 

Table 4 presents the correlation matrix (all *p* values are <0.05 unless stated otherwise). Psychological risk was negatively correlated with brand trust (r = −0.233, *p* < 0.05) and nutritional disclosure (r = −0.188, *p* < 0.05). Nutritional disclosure was positively correlated with brand trust (r = 0.485) and price fairness (r = 0.297, *p* < 0.05). Price fairness was positively correlated with brand trust (r = 0.519, *p* < 0.05). Eco-friendly packaging was positively correlated with brand trust (r = 0.490, *p* < 0.05), price fairness (r = 0.457, *p* < 0.05), and nutritional disclosure (r = 0.353, *p* < 0.05). Means and standard deviations are displayed in Table 4 for brand trust, price fairness, nutritional disclosure, psychological risk, and eco-friendly packaging.

### 4.3. Results of Hypothesis Testing

Table 5 shows the results of Hayes’ PROCESS macro model 7. Models 1 and 2 include price fairness and brand trust as the dependent variables. Both models were statistically significant. Psychological risk had negative impacts on price fairness (β = −0.627, *p* < 0.05) and brand trust (β = −0.262, *p* < 0.05). Also, price fairness positively affected brand trust (β = 0.597, *p* < 0.05). Nutritional disclosure significantly moderated the effect of psychological risk on price fairness (Psychological risk × Nutritional disclosure (β = 0.172, *p* < 0.05)). Additionally, the index of mediated moderation effect appeared significant. In summary, all hypotheses were supported. 

**Figure 3:**
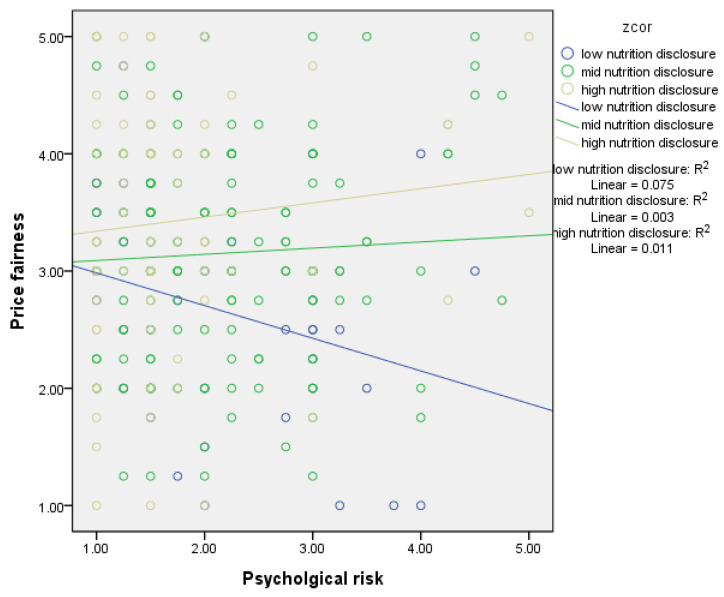
Moderating effect of nutritional disclosure on the relationship between psychological risk and price fairness.

Figure 3 illustrates the moderating effect of nutritional disclosure on perceived price fairness based on a simple slope method. Based on the magnitude of the conditional effect of the focal predictor, only the high group showed the positive and significant effect of psychological risk on price fairness. The yellow line in Figure 3 presents the slope of the high-nutrition group. 

Table 6 shows additional results of Hayes’ PROCESS macro model 7. Models 3 and 4 include price fairness and brand trust as the dependent variable, respectively. Both models were statistically significant. Eco-friendly packaging had a positive impact on brand trust (β = 0.346, *p* < 0.05), and price fairness positively affected brand trust (β = 0.433, *p* < 0.05). Nutritional disclosure significantly moderated the effect of eco-friendly packaging on price fairness (β = 0.093, *p* < 0.05). Also, the index of the mediated moderation effect was not significant.

**Figure 4:**
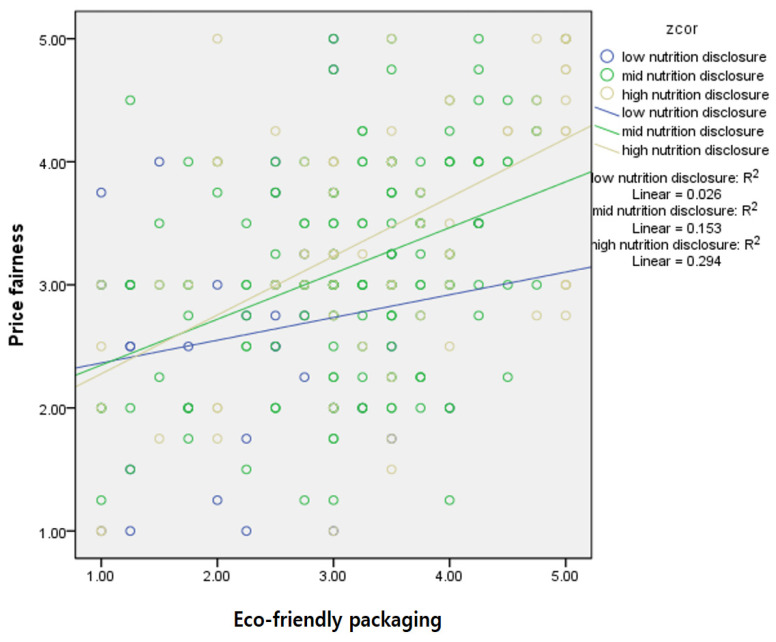
Moderating effect of nutritional disclosure on the relationship between eco-friendly packaging and price fairness.

Figure 4 shows the moderating effect of nutritional disclosure on perceived price fairness based on the simple slope method. The slopes for high, middle, and low groups of nutritional disclosures appeared as yellow, green, and blue, respectively. The results indicated that the effect of eco-friendly packaging on price fairness was the strongest in the case of the high-nutrition disclosure group. 

## 5. Discussion

This work explored consumer perceptions of bottled water. The perceived psychological risk was low, whereas the nutritional disclosure rating was relatively high. The first goal of this work was to investigate the relationships among psychological risk, price fairness, and brand trust. The results showed that psychological risk negatively affected price fairness; that is, actual consumer experience differed from consumer expectations of bottled water, negatively affecting price-fairness perceptions. It is aligned with the prior studies’ arguments that individuals dislike uncertainty in their decision-making [50,51]. In addition, psychological risk negatively impacted brand trust; the discrepancy in expectations versus actual bottled water consumption experience lowered the level of trust in branded bottled water. Thus, psychological risk is detrimental to both perceptions of price fairness and brand reputation. Moreover, there was a positive effect of price fairness on brand trust; when consumers perceive the price of bottled water as rational, they are more likely to view the product as credible. In addition, eco-friendly packaging was found to play a pivotal role in brand trust, but it alone appears insufficient to improve the price-fairness perceptions of consumers. It may be that packaging is only an “auxiliary” factor in the decision to consume branded water. 

Another aim of this study was to examine the moderating effect of nutritional disclosure on the relationship between psychological risk and price fairness. Greater nutritional disclosure might reduce the negative effect of psychological risk on price-fairness perceptions. In the high nutritional disclosure group, perceptions of price fairness differed little between the low and high psychological risk subgroups. However, the low nutritional disclosure group presented a relatively stronger perception change of price fairness between low psychological risk and high psychological risk negatively because of the reduced value from 3.07 to 2.78. We found a significant moderating effect of nutritional disclosure on the relationship between eco-friendly packaging and price fairness. The results suggest that consumers are more willing to pay for bottled water products with packaging that focuses on protecting the environment and providing product information. Plus, the results showed the index of mediated moderation effect appeared significant only in the case of psychological risk as an independent variable. However, the mediated moderation impact was not significant in the case of eco-friendly packaging. 

## 6. Conclusions

### 6.1. Theoretical and Managerial Implications 

This work sheds light on the relationships among price fairness, psychological risk, and brand trust, building on studies that have explored the relationship between psychological risk and brand trust [34,37] and the impact of price fairness on brand trust [38,39,52]. This research also offers theoretical value by clarifying the significant effect of psychological risk on price fairness, a relationship that has been insufficiently explored in the existing literature. Given the limited empirical investigation of this connection, the findings of this study are particularly valuable. Additionally, the research contributes to the literature by addressing the gap regarding the link between psychological risk and price fairness within the context of the bottled water industry. Moreover, this study advances understanding by revealing the moderating role of nutritional disclosure in the relationship between psychological risk and price fairness. We also identify the moderating effect of nutritional disclosure on the association between eco-friendly packaging and price fairness. Finally, this work contributes to the literature by highlighting the significance of mediated moderation effects in the context of psychological risk, offering a deeper understanding of the interplay between five key attributes: psychological risk, eco-friendly packaging, price fairness, nutritional disclosure, and brand trust. 

This research has practical implications for bottled water companies. First, managers of bottled water businesses might be able to focus on consumer expectations of their products to minimize the gap between expectations and experience. This could improve price-fairness perceptions and brand reputation. In other words, the managers might need to allocate their resources to maintain the quality of products, which contains the packaging, storage, delivery, and the water itself. Moreover, managers of bottled water businesses might refrain from frequently changing the product price because doing so is likely to undermine perceptions of price fairness, which might be harmful to the brand’s credibility. Finally, managers could allot more of their budgets to the provision of nutrition information (e.g., improving packaging designs to enhance the visibility of nutrition information). Such efforts could enhance consumers’ perception of price fairness by fostering a transparent image of the business within the bottled water industry. Additionally, investing more resources in eco-friendly packaging may strengthen brand reputation and positively influence perceptions of price fairness and sustainability. Consumers are more likely to feel confident about the safety of bottled water when eco-friendly packaging is used, as it helps mitigate concerns related to environmental issues, such as microplastics.

### 6.2. Limitations and Suggestions for Future Research 

This research had some limitations. First, we only studied the Dasani brand. Other brands should be considered in future work. This study also focused on four key variables; however, future research could explore additional factors to gain a more comprehensive understanding of bottled water consumers. Using advanced statistical techniques, such as structural equation modeling, could provide deeper insights into the relationships among these variables. Furthermore, this research relied solely on survey data for data collection. Future studies could benefit from employing a broader range of research methods, such as experiments, longitudinal studies, or scenario-based approaches, to enhance the robustness and generalizability of the findings.

## Figures and Tables

**Table 1:** Measurement items in this study.

Variable	Code	Item
Brand trust	BT1	I trust the brand Dasani.
	BT2	Dasani is reliable.
	BT3	Dasani is credible.
	BT4	Dasani is trustworthy.
Price fairness	PF1	The price of Dasani goods is fair.
	PF2	The price of Dasani goods is rational.
	PF3	The price of Dasani goods is reasonable.
	PF4	The price of Dasani goods is acceptable.
Nutritional disclosure	ND1	Dasani offers ingredient information.
	ND2	Dasani discloses its ingredients.
	ND3	Dasani water is presented well.
	ND4	Dasani provides me with ingredient information.
Psychological risk	PR1	Dasani is psychologically risky.
	PR2	Dasani is risky to consume.
	PR3	Dasani causes uncertainty.
	PR4	Dasani did not meet my expectations.
Eco-friendly packaging	EP1	Dasani packaging is environmentally friendly.
	EP2	Dasani packaging is eco-friendly.
	EP3	Dasani packaging is recyclable.
	EP4	Dasani packaging is useful to reduce plastic garbage.

**Table 2:** Participant demographics (N = 308).

Demographics	Frequency	Percentage
Male	84	27.3
Female	224	72.4
Employed	212	68.8
Unemployed	96	31.2
20 s	57	18.5
30 s	106	34.4
40 s	104	33.8
50 s	33	10.7
≥60 years old	8	2.6
Monthly household income	98	31.8
<$2500	105	34.1
$2500–$4999	57	18.5
$5000–$7499	48	15.6
>$7500		

**Table 3:** Results of exploratory factor analysis and reliability testing.

Variable	Code	Loading	Cronbach’s α	Eigenvalue	Variance Explained (%)
Brand trust	BT1	0.825	0.950	7.699	38.495
	BT2	0.842			
	BT3	0.835			
	BT4	0.832			
Price fairness	PF1	0.825	0.930	3.295	16.475
	PF2	0.852			
	PF3	0.886			
	PF4	0.877			
Nutritional disclosure	ND1	0.884	0.916	2.163	10.816
	ND2	0.904			
	ND3	0.716			
	ND4	0.911			
Psychological risk	PR1	0.905	0.865	1.347	6.737
	PR2	0.923			
	PR3	0.882			
	PR4	0.600			
Eco-friendly packaging	EP1	0.865	0.882	1.593	7.967
	EP2	0.876			
	EP3	0.634			
	EP4	0.840			

Note: total variance explained = 80.49%.

**Table 4:** Correlation matrix.

Variable	Mean	SD	1	2	3	4	5
Brand trust	3.508	1.093	1				
Price fairness	3.152	0.941	0.519 *	1			
Nutritional disclosure	3.804	0.899	0.485 *	0.297 *	1		
Psychological risk	1.995	0.918	−0.233 *	−0.024	−0.188 *	1	
Eco-friendly packaging	3.115	1.009	0.490 *	0.457 *	0.353 *	0.063	

Note: * *p* < 0.05; SD, standard deviation.

**Table 5:** Results of hypothesis testing for psychological risk.

	Model 1 Price Fairness		Model 2 Brand Trust	
	β	t-value	β	t-value
Constant	3.236	6.14 *	2.149	9.99 *
Psychological risk	−0.627	−2.74 *	−0.262	−4.65 *
Nutritional disclosure	−0.030	−0.23		
Interaction	0.172	2.98 *		
Price fairness			0.597	10.86 *
F-value	13.15 *		71.10 *	
R^2^	0.1149		0.3180	
Conditional effect of the focal predictor	β	t-value		
Nutritional disclosure				
3.00 (Low)	−0.109	−1.48		
4.00 (Mid)	0.063	1.10		
5.00 (High)	0.235	2.67 *		
Mediated moderation effect	Index	LLCI	ULCI	
	0.1031 *	0.0282	0.1805	

Note: * *p* < 0.05; Interaction: psychological risk × nutritional disclosure (test of unconditional interaction: F = 8.91); LLCI, lower limit confidence interval; ULCI, upper limit confidence interval.

**Table 6:** Results of hypothesis testing for eco-friendly packaging.

	Model 3 Price Fairness		Model 4 Brand Trust	
	β	t-value	β	t-value
Constant	2.363	4.37 *	1.063	5.38 *
Eco-friendly packaging	0.025	0.01	0.346	6.15 *
Nutritional disclosure	−0.092	−0.67		
Interaction	0.093	2.01 *		
Price fairness			0.433	7.19 *
F-value	31.99 *		82.17 *	
R^2^	0.2400		0.3502	
Conditional effect of the focal predictor	β	t-value		
Nutritional disclosure				
3.00 (Low)	0.281	4.13 *		
4.00 (Mid)	0.374	7.52 *		
5.00 (High)	0.467	6.90 *		
Mediated moderation effect	Index	LLCI	ULCI	
	0.0404	−0.0072	0.0893	

Note: * *p* < 0.05; Interaction: eco-friendly packaging × nutritional disclosure (test of unconditional interaction: F = 4.06); LLCI, lower limit confidence interval; ULCI, upper limit confidence interval.

## Data Availability

The data presented in this study are available upon request from the corresponding author (The data are not publicly available due to privacy).
